# A novel *Enterococcus durans* with antimicrobial, anti-diabetes and anti-alzheimer activities isolated from Egypt

**DOI:** 10.1186/s13568-025-01836-2

**Published:** 2025-03-26

**Authors:** Hussam H. Arafat, Mahmoud A. Shoulkamy, Mohamed M. Imam, Amany M. A. Ali

**Affiliations:** Department of Botany and Microbiology, Faculty of Science, Minia University, Minia City, 61519 Egypt

**Keywords:** *Enterococcus*, Antimicrobial activity, Anti-diabetes, Anti-Alzheimer

## Abstract

*Enterococcus* sp. is a subset of lactic acid bacteria that have beneficial effects on human health including prevention of gut chronic diseases, controlling Alzheimer and diabetes. The study involves morphological and biochemical identification of *Enterococcus* sp. and 16S rRNA gene sequencing methods, alongside an exploration of it’s antimicrobial, anti-diabetes and anti-Alzheimer efficacy. The cell-free supernatant (CFS) derived from this isolate (*Enterococcus durans*) exhibited a significant antibacterial activity against both Gram-positive (*Bacillus subtilis, Staphylococcus aureus, Enterococcus faecalis*) with inhibition zones of 31.1, 33 and 27 mm, respectively and Gram-negative (*Escherichia coli, Klebsiella pneumoniae, Salmonella typhi*) bacteria, recording inhibition zones of 24, 25 and 30 mm, respectively. The least values of MIC and MBC were 15.62 µg/ml and 31.25 µg/ml, respectively; in case of *Bacillus subtilis* and *Staphylococcus aureus*. The highest MIC and MBC were 62.5 µg/ml and 250 µg/ml respectively, when testing CFS against *Klebsiella pneumonia*. Notably, the stability of CFS was maintained at various temperatures, including autoclaving conditions (121 ℃). The isolate displayed tolerance across a wide pH range (2.5—9.5), with enhanced activity observed at acidic pH levels. Butyrylcholinesterase inhibition was estimated to be 84.6%, while amylase inhibition was 97.6% & 94.2%, respectively. GC–MS revealed metabolites not defined previously in enterococci: 1H-purin-6-amine, [(2-fluorophenyl) methyl]—(29.72%), hexadecanoic acid, 2, 3 dihydroxypro polyester (18.60%), oleic acid (11.60%) and 9-octadecenamide (6.54%). Hence, our strain is a reservoir of strong bioactive compounds, with antimicrobial, anti-diabetes and anti- Alzheimer potentials.

## Introduction

Lactic acid bacteria (LAB) are Gram-positive, non-motile, catalase-negative, and non-spore-forming microorganisms with the ability to produce lactic acid. LAB exhibit probiotic properties, including adhesion and colonization in the digestive mucosa, vitamin production, and the synthesis of antimicrobial compounds (Ananou et al. 2007; Oliveira et al. 2008; LeBlanc et al. 2011). Notably, LAB demonstrate the inhibition of various bacteria, such as *Escherichia*, *Staphylococcus*, *Salmonella*, *Shigella*, and *Bacillus*, along with antifungal activity against *Candida* sp. (Islam et al. 2020; Adikari et al. 2021). Various genera of cocci lactic acid bacteria, encompassing *Pediococcus*, *Leuconostoc*, *Weissella*, *Lactococcus*, *Enterococcus*, and *Streptococcus* (Whitman et al. 2015), exhibit distinctive characteristics. LAB are Generally Recognized as Safe (GRAS) and capable of synthesizing exopolysaccharides (EPS) that are often used in the manufacture of fermented milks to improve their texture. They have been used in the elaboration of low-fat cheeses (mozzarella) to increase moisture retention in low-fat mozzarella (Spencer and de Spencer 2001).

Enterococci in particular are highly tolerant to salt, acidic pH, heat and bile salts, their natural habitats are the gastrointestinal tract of humans and animals, vegetables, plant material, and other food (Whitman et al. 2015). In manufacturing of raw milk cheese, *Enterococcus* sp. could decrease* Listeria monocytogenes* counts (Nunez et al. 1997) and completely inactivated the pathogen during ripening of cheese even without starter culture (RodrÍGuez et al. 2001). Many enterococci like *E. durans*, *E. faecalis* and *E. faecium*, have been described in artisanal cheeses, where their role in flavor development has been documented (Dapkevicius et al. 2021). Enterococci have taken a great attention being an important group of LAB with various benefits though they were known as common pathogens (Franz et al. 2011). Furthermore, they may produce antibacterial peptides (bacteriocins), known as enterocins, which can affect products’ shelf life and safety (Schirru et al. 2012). Strains of *E. faecium* and *E. faecalis* have also been used as probiotics for farm animals and humans (Semedo et al. 2003).

While nisin, a widely used bacteriocin, serves as a food bio-preservative, its limitations, including decreased stability and a narrow pH range (5.0–7.0), with only slight effects on gram-negative bacteria, prompt the exploration of new antimicrobial components with a broad spectrum (Héchard and Sahl 2002). The safety and efficiency of antimicrobial components derived from lactic acid bacteria, including enterococci, have garnered considerable attention in recent research as potential natural alternatives to antibiotics and chemical preservatives in the food industry (Bhakta et al. 2023).

The use of probiotic strains in treatment of infectious diseases is considered both safe and stable, avoiding an increase in the risk of multi-drug resistance among pathogens (Roghmann and McGrail 2006). Lactic bacteria can contribute to therapy of other diseases. Alzheimer’s disease (AD) is one of the most common neurodegenerative diseases that appear among the elderly people, accompanied by dementia in AD patients. The imbalance in intestinal microflora, or reduction in number of beneficial bacteria, is always observed in patients with AD. This leads to inflammation, insulin resistance, glucose metabolism dysfunction, like the onset of AD and other disorders (Qu et al. 2024). The medication rivastigmine is administered to treat and manage neurodegenerative disorders like dementia in AD patients. This drug is a cholinesterase inhibitor. Researchers found that rivastigmine had the highest rate of gastrointestinal side effects like vomiting and nausea. Probiotics were tested as alternatives to these drugs. *Streptococcus, Bifidobacterium* and *Lactobacillus* have recently been studied as anti-AD (Patel and Gupta 2023). The probiotics supplementation improved spatial memory and learning in AD rat model (Athari Nik Azm et al. 2018). Administration of probiotics suppressed neuroinflammation AD-injected mice (Li et al. 2018).

Another widely spread disease is diabetes. Inhibiting activities of carbohydrate digestion enzymes to delay glucose absorption is applied for effective treatment of hyperglycemia. High α-amylase inhibitory activity or α-glucosidase inhibitors are related to undesired side effects (Hui et al. 2020). The α-glucosidase inhibitors, including acarbose, miglitol, and voglibose, have been used for managing hyperglycemia and T2DM (Type 2 diabetes). These medications have been reported to elicit gastrointestinal side effects, such as diarrhea and flatulence, due to prolonged inhibition of starch hydrolysis (Oboh et al. 2016). Studies have demonstrated that diabetes patients (T2DM) have an increased risk of dementia and cognitive impairment (Ahtiluoto et al. 2010). the risk of dementia occurs through several possible mechanisms, including insulin resistance and oxidative stress (Whitmer 2007). Oxidative stress accumulates β-amyloid plaques in the brain, leading to AD (Reddy et al. 2009). Probiotics are regarded as potential biotherapeutics for T2DM (Panwar et al. 2013). Administration of probiotic bacterial strains *Enterococcus* sp., lowered blood glucose levels in diabetic rats (Wei et al. 2020). Diabetic rats treated with the probiotic bacteria showed asignificant decrease in cholesterol and triglycerides (Baynes 1991).

Thus, our study aimed to investigate enterococci with probiotic features like tolerance to bile salts and acidity, efficiency against different bacteria, and stability of these antimicrobial components that have a potential as natural alternatives to antibiotics and chemical preservatives in the food industry. This work also involves detecting anti-diabetic and anti- Alzheimer activity of the tested strain.

## Materials and methods

### Isolation and morphological identification of lactic acid bacteria (LAB)

Yoghurt samples were purchased from dairy companies and 10^–1^ dilution of selected samples was prepared by diluting 1 gm of yoghurt in approximately 10 ml sterile distilled water (SDW). Each diluted sample (0.5 ml) was plated on de Man, Rogosa and Sharpe (MRS) agar plates with the following constituents (in L): glucose (20), peptone (10 g), yeast extract (5 g), beef extract (5 g), sodium acetate (5 g), K_2_HPO_4_ (2 g), ammonium citrate (2 g), MgSO_4_.7H_2_O (0.2 g), MnS0_4_.4H_2_O (0.05 g), Tween-80 (1 ml) and agar (20 g) (de Man et al. 1960) and incubated under aerobic conditions in a static incubator at 37 ℃ for 3 days.

Sixteen white, convex or raised colonies, with smooth surfaces and diameters (≤ 2 mm), were selected for purification (on MRS agar) and Gram staining (Grange and Lyne 2004).

### Preservation of the isolate

Short-term storage (for 1 month at 4 °C) involved three methods: agar slant, stab inoculation using semi-solid MRS medium (with 0.3% CaCl_2_ as pH neutralizer) as described by Björkroth and Holzapfel (2006), and inoculation of MRS broth with young bacterial culture (not followed by incubation). For long-term storage, this isolate was maintained as glycerol stocks at -20 °C (Spencer and de Spencer 2001).

### Motility test

Stab inoculation in tubes of semi-solid MRS medium was performed as described by MacFaddin (2000) and motility was assessed after incubation at 37 °C for 48 h (MacFaddin 2000).

### Biochemical characterization

Various biochemical tests were conducted in MRS broth with bromothymol blue if needed (Somasegaran and Hoben 1994), including gas production (CO_2_) (Schillinger and Lücke 1987), catalase production (Kozaki et al. 1992), gelatin hydrolysis (Aneja 2007), starch hydrolysis on agar plates (Forouhandeh et al. 2010), tryptophanase activity (Kovacs 1928), nitrate reduction (Reddy et al. 2007), citrate utilization (Mithun et al. 2015), hydrolysis of arginine (Samelis et al. 1994), NaCl tolerance (Ni et al. 2015), growth at acidic and alkaline pH (Ni et al. 2015), growth at different temperatures (Samelis et al. 1994), Voges-Proskauer test (Barritt 1936), production of dextran (slime) from sucrose (Hitchener et al. 1982), production of hydrogen sulfide (H_2_S) (Shay and Egan 1981), methylene blue reduction (Abanoz and Kunduhoglu 2018), carbon source utilization (Abanoz and Kunduhoglu 2018), urease test (Steadham 1979) and bile salts tolerance (Menconi et al. 2014).

### Amplification of 16S rRNA gene

Genomic DNA was extracted as described by Spencer and de Spencer (2001), but we omitted steps of isopropanol and proteinase K. Firstly, lysis buffer containing lysozyme (Geneaid, Taiwan) was added to young cultures and microfuge tubes were incubated at 37 °C in water bath for 2 h. Then, we added 50 µl of 10% SDS, sodium chloride and µl of chloroform–isoamyl alcohol (24: 1). The upper phase obtained after centrifugation was transferred to a fresh tube. Two volumes of chilled 100% ethanol were added to precipitate nucleic acids, stored at -20 °C overnight, and then centrifuged at 12,000 g for 10 min. Pellets were washed twice with 70% ethanol chilled at -20 °C and finally air-dryed. DNA was resuspended in 50 µl of TE (Tris- EDTA) buffer and stored at 4 °C (Spencer and de Spencer 2001).

PCR amplification was performed using the universal primers: the forward primer 27 F (metabion international AG) sequence is: (5′-AGAGTTTGATCCTGGCTCAG-3′) and the reverse primer 1492 R (metabion international AG) sequence is (5′-GGTTACCTTGTTACGACTT-3′). The master mix was 2 × Taq PCR master mix (Biomatik)**.** The size of the reaction was 50 µl (2 µl forward primer, 2 µl reverse primer, 6 µl genomic DNA, 25 µl master mix, 15 µl free nuclease water). The PCR was carried out by Gene AMP PCR System 9700 from PE Applied Biosystems (Perkin Elmer, Ohio, USA). The PCR program for 16S rRNA included an initial denaturation stage at 95 ºC for 2 min. followed by 35 cycles of the second stage which involved three steps: i) Denaturation at 95 ºC for 1 min, ii) Primer annealing at 52 ºC for 1 min and iii) Extension at 72 ºC for 1 min. The third stage has a final extension of 72 ºC for 10 min. The PCR product was sequenced using DNA Sequencing Services by Sigma Company in South Korea. Amplification was confirmed by agarose gel (0.8%) electrophoresis in TAE buffer. Size of the gene was determined compared to Mid-Range DNA Ladder (100 bp to 3 kb, "Jenabioscience"). The PCR product (1.5 kb) was purified and then sequenced using DNA Sequencing Services by Sigma Company in South Korea. Finch TV Software was used to edit the raw sequence data. BLAST analysis was conducted to determine sequence similarities between isolated strains and sequences of known bacterial species deposited in Gene Bank was done by the help of NCBI database (https://blast.ncbi.nlm.nih.gov/Blast.cgi). Our isolate was very similar to *Enterococcus durans* strain JCM 8725 (NR_113257.1).

### Antimicrobial activity test

The well diffusion method was employed to test antimicrobial activity against various indicator organisms (Sonbol et al. 2020). The selected isolate was grown in MRS broth for 5 days/37 °C, and cell-free supernatant (CFS) was obtained by centrifugation at 6000 rpm/10 min. The final pH was found to be 4.5. Thus, we compared the antimicrobial efficiency of CFS (crude) and sterile MRS (at pH 4.5) against the chosen indicator *Staphylococcus* sp*.* Organic matabolites were extracted by mixing ethyl acetate with CFS (1:1) in the separation funnel. Ethyl acetate layer was then air dried to remove ethyl acetate that is highly volatile. 50 mg of the sample were dissolved in 1.0 ml diluent. The sample and the diluent were tested against various indicators. The antibiotic gentamycin (1 mg/ml) was also tested as a positive control.

### Determining minimal inhibitory concentration (MIC) & minimal bacteriocidal concentration (MBC)

Stock Solution 10 mg of ethyl acetate extract was dissolved in 10 ml distilled water (1000 µg/ml). MICs have been determined using concentrations derived traditionally from serial twofold dilutions indexed to the base 2 (eg, 125, 250, 500, 1000 µg/ml). The plastic microdilution trays (with 96 wells) were inoculated with the indicator bacteria (10 μl) in 0.1 ml antimicrobial agent solution. Negative controls without inoculation by test organisms) were included, and positive controls (no antimicrobial agent) were plated and used to establish a baseline concentration of the microorganism used. The amount of growth in the wells containing the antimicrobial agent with the amount of growth in the positive-control wells were compared when determining the growth end points. Minimal Inhibitory Concentration (MIC) & Minimal Bacteriocidal Concentration (MBC) were determined after incubation (35±2°C for 16 to 20 hours) in an ambient air incubator, and the turbidity was detected using BioTek 800 TS microplate reader at OD= 630 nm (Wikler 2006). To determine the MBC, the dilution representing the MIC and at least two of the more concentrated test dilutions were plated and enumerated to determine viable CFU/ml. The MBC is the lowest concentration that demonstrates a pre-determined reduction (such as 99.9 %) in CFU/ml when compared to the MIC dilution. Results were represented as µg/ml.

### Thermal and pH stability of antimicrobials in cell-free supernatant (CFS)

In this experiment, the cell-free supernatant (CFS) was directly subjected to different temperatures and autoclaving, as well as various pH levels (i.e.: the filtrate wasn’t subjected to ethyl acetate extraction). *Staphylococcus* sp. was chosen as an indicator to assess activity of antimicrobials in cell-free supernatant (Oliveira et al. 2008; Abanoz and Kunduhoglu 2018).

### Butyryl cholinesterase activity assay and inhibition studies

The following chemicals were obtained from Biodiagnostic: BChE from equine serum, butyrylthiocholine iodide and 5, 5′-dithiobis-bis-nitrobenzoic acid (DTNB). The absorption was read immediately at 405 nm on a microplate reader. The concentration of the test compound required to inhibit BuChE activity by 50% (IC_50_) was calculated using an enzyme inhibition dose response curve (Gorun et al. 1978).

### In vitro α-amylase inhibition (anti-diabetes activity)

The α-amylase inhibition assay was performed using the 3,5-dinitrosalicylic acid (DNSA) method (Wickramaratne et al. 2016).

### Gas chromatography–mass spectrometry (GC–MS) analysis

The chemical composition of our sample was performed using Trace GC1310-ISQ mass spectrometer (Thermo Scientific, Austin, TX, USA) with a direct capillary column TG–5MS (30 m × 0.25 mm × 0.25 µm film thickness). The column oven temperature was initially held at 35 °C and then increased by 3 °C /min to 200 °C hold for 3 min. increased to the final temperature 280 °C by 3 °C /min and hold for 10 min. The components were identified by comparison of their retention times and mass spectra with those of WILEY 09 and NIST 11 mass spectral database (Huwaimel et al. 2023). The compounds contributing to the biological activities (antimicrobial, anti-diabetes, and anti-Alzheimer) were primarily identified based on their abundance in the ethyl acetate extract, as determined by GC–MS analysis. The selection of key compounds, such as 1H-Purin-6-amine, [(2-fluorophenyl) methyl], hexadecanoic acid, 2,3-dihydroxypropyl ester, oleic acid, and 9-octadecenamide, was guided by their relative area percentage and previously reported bioactivities in the literature. For instance, 1H-Purin-6-amine is known for its antimicrobial, anti-inflammatory, and enzyme inhibitory properties, including α-amylase inhibition; hexadecanoic acid, 2,3-dihydroxypropyl ester has been reported for its antibacterial and anticancer potential; oleic acid is correlated with anti-inflammatory and neuroprotective properties; and 9-octadecenamide exhibits strong antioxidant, antibacterial, and hypolipidemic effects. These compounds were highlighted as key contributors due to their known bioactivities, which align with the observed biological effects of the extract.

### Data statistical analysis

Data are presented as mean ± SE by applying the SAS program (version 9.4, 2013). Duncan’s test was used to determine the significance of the mean differences. The probability was considered significant at p < 0.05.

## Results

### Isolation, biochemical characterization and molecular identification of *Enterococcus* sp.

Colonies of *Enterococcus durans* exhibited circular, convex morphology, translucent, with an off-white color, smooth surface, and entire margin. Under microscopic examination, the strain appeared oval in chains, Gram-positive, and non-spore former. It exhibited catalase negativity and acid production from glucose without gas formation. Negative results were also observed for motility, indole, nitrate reduction, urease, starch and gelatin hydrolysis, and H_2_S production. Positive results were recorded for acetoin production, slime formation, methylene blue reduction, milk coagulation, arginine hydrolysis, and growth in bile salts up to 40%.

Positive carbon source utilization was observed for lactose, mannose, fructose, galactose, cellobiose, mannitol, ribose, raffinose, glycerol, rhamnose, and maltose. However, negative results were observed for citrate and arabinose. Growth occurred at temperatures ranging from 5 to 45 °C, where good growth was reached after 2–3 days (at 30- 45 °C) and 14 days at 5 °C. Tolerance to salinity was up to 6.5% and pH levels were between 4.5 and 9.5. Highly acidic levels (pH 3 or 3.5) rendered growth.

The amplified 16S rRNA gene, visualized by agarose gel electrophoresis, exhibited a size of approximately 1.5 kbp. The isolate identity (99.91%) is very close to *Enterococcus durans* strain JCM 8725 NR_113257.1.

 The sequence was deposited in GenBank with accession number (OP648139) with the name *Enterococcus durans* strain AMA1. The isolate was then deposited in the Culture Collection, Ain Shams University (CCASU) of the World Data Centre for Microorganisms (WDCM) under a specific code (*Enterococcus durans*, CCASU-2023–61, (https://doi.org/10.12210/ccinfo.1186).

### Antimicrobial activity of *Enterococcus durans*, minimal inhibitory concentration (MIC) & minimal bacteriocidal concentration (MBC)

**Fig. 1:**
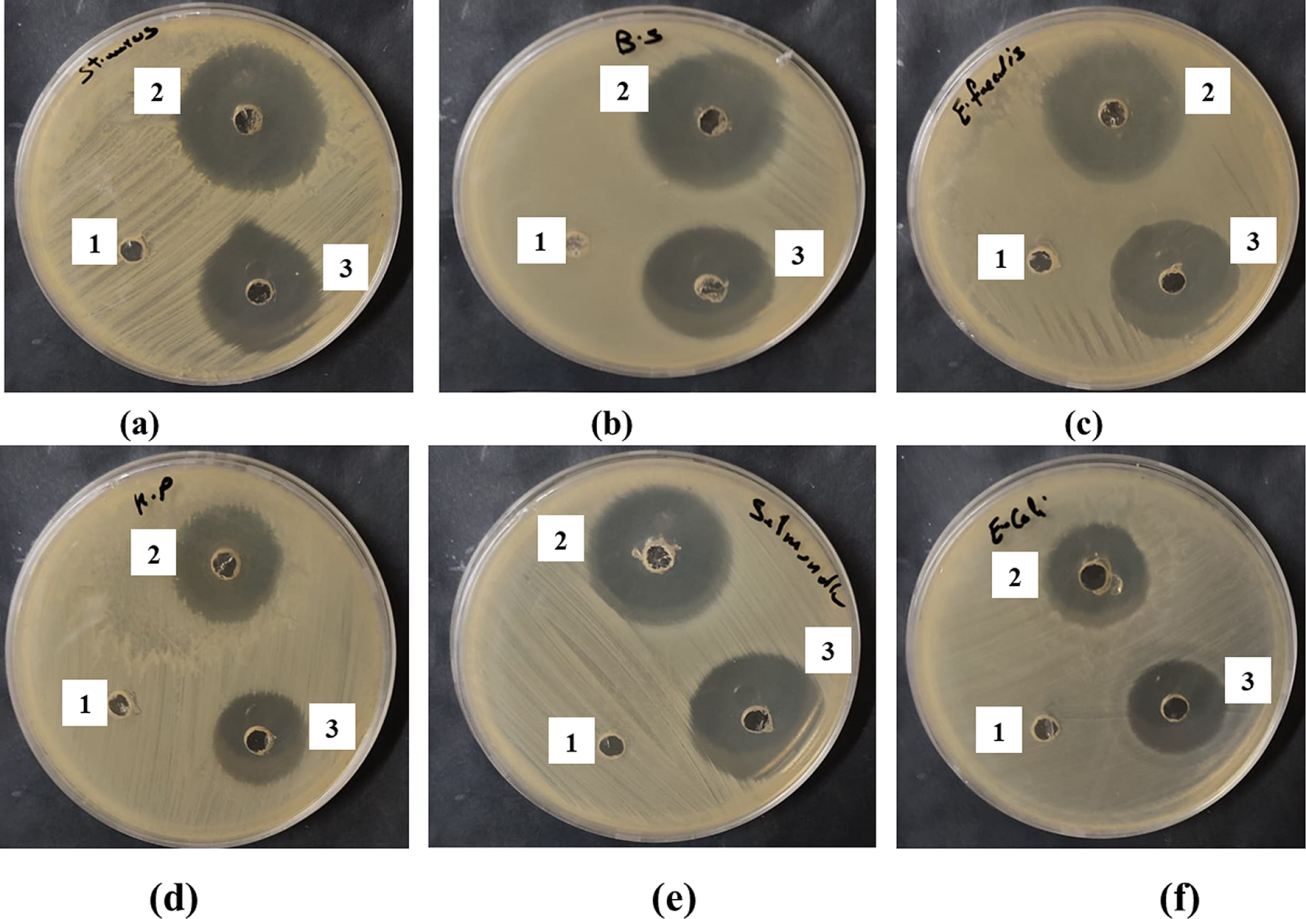
Antimicrobial activity of ethyl acetate extract of *Enterococcus durans* strain AMA1. *(**a**) = *Staphylococcus aureus*, (**b**) = *Bacillus subtilis*, (**c**) = *Enterococcus faecalis*, (**d**) = *Klebsiella pneumonia*, (**e**) = *Salmonella typhi*, (**f**) = *Escherichia coli*. *1 = Control (diluent only)*,* 2 = Extract of *E. durans* strain AMA1, 3 = Gentamycin.

The isolated strain exhibited a broad spectrum antagonistic activity against both Gram-positive (*Bacillus subtilis, Staphylococcus aureus, Enterococcus faecalis*) with inhibition zones of 31.1, 33 and 27 mm, respectively and Gram-negative (*Escherichia coli, Klebsiella pneumoniae, Salmonella typhi*) bacteria, recording inhibition zones of 24, 25 and 30 mm, respectively (Table 1 and Fig. 1).

**Table 1:** Antimicrobial activity, MIC and MBC of ethyl acetate extract from *Enterococcus durans* strain AMA1

Indicator bacteria	Inhibition zone		MIC (µg/ml)	MBC (µg/ml)
	Sample	Control		
*Bacillus subtilis* (ATCC 6633)	31 ± 0.1	29 ± 0.2	15.62	31.25
*Staphylococcus aureus* (ATCC 6538)	33 ± 0.1	27 ± 0.2	15.62	31.25
*Enterococcus faecalis* (ATCC 29212 )	27 ± 0.2	30 ± 0.1	31.25	62.5
*Escherichia coli* (ATCC 8739)	24 ± 0.1	24 ± 0.1	62.5	125
*Klebsiella pneumoniae* (ATCC13883)	25 ± 0.2	22 ± 0.1	62.5	250
*Salmonella typhi* (ATCC 6539)	30 ± 0.1	31 ± 0.2	31.25	62.5

*Sample = Ethyl acetate extract from *E. durans* strain AMA1,

Control = Gentamycin, Inhibition zone (mm)

The least values of MIC and MBC were 15.62 µg/ml and 31.25 µg/ml, respectively; in case of *Bacillus subtilis* (ATCC 6633) and *Staphylococcus aureus* (ATCC 6538). The highest MIC and MBC were 62.5 µg/ml and 250 µg/ml respectively, when testing cell-free supernatant (CFS) against *Klebsiella pneumoniae* (ATCC13883). Table (1) illustrates the obtained MIC and MBC values of CFS from *E. durans.*

### Thermal and pH stability of antimicrobials in cell-free supernatant (CFS)

**Fig. 2:**
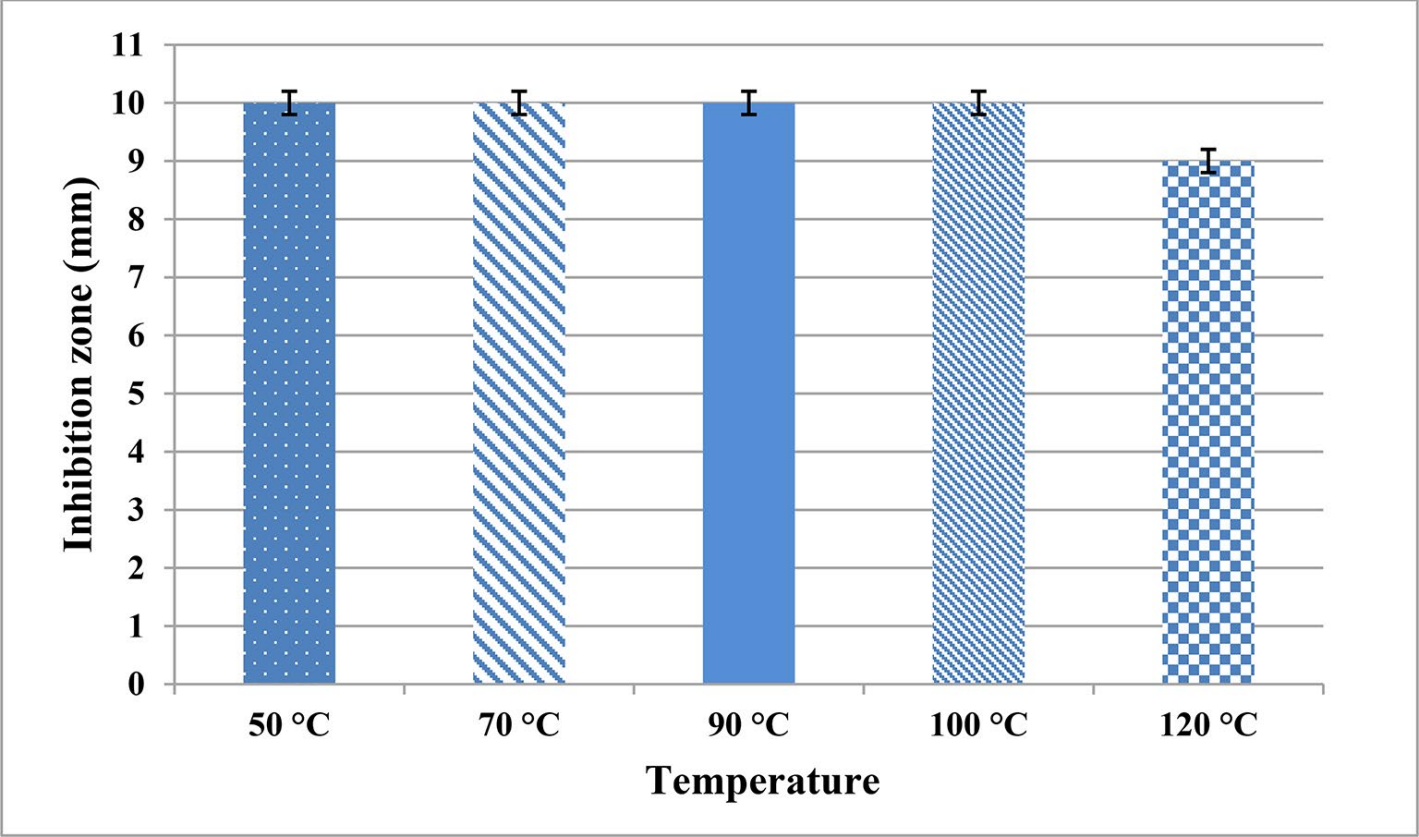
Thermal stability of antimicrobials in Cell-Free Supernatant (CFS) of *Enterococcus durans* strain AMA1

**Fig. 3:**
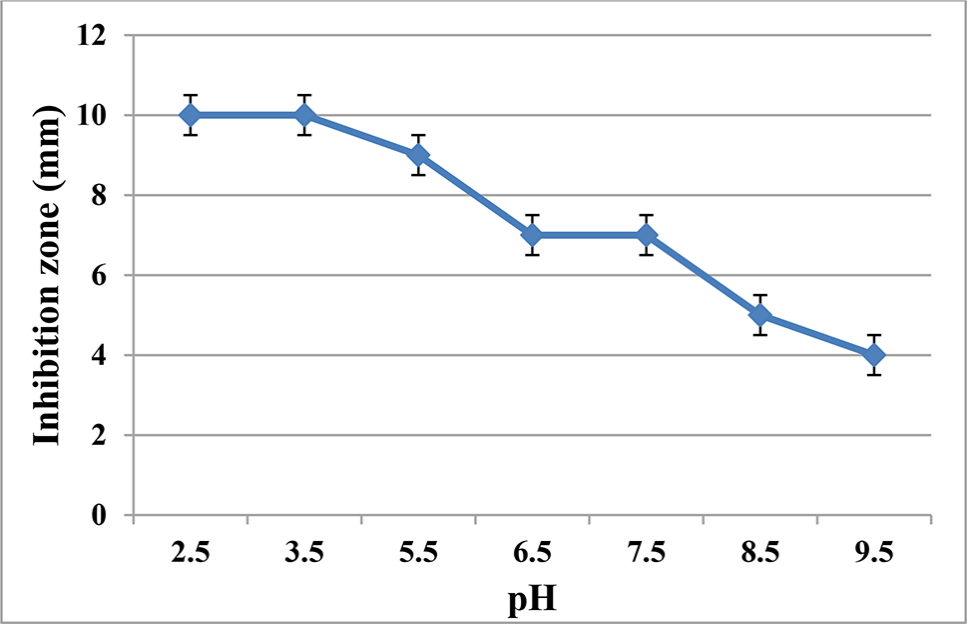
pH stability of antimicrobials in Cell-Free Supernatant (CFS) of *Enterococcus durans* strain AMA1

Figures 2 & 3 illustrate the impact of temperatures and pH on the cell-free supernatant (CFS) of our culture. Supernatant remained active at temperatures ranging from 50 ºC to 100 ºC, and even after autoclaving (121 ºC for 15 min); it produced an inhibition zone of approximately 10 mm (Fig. 2). Though heating didn’t cause a notable decrease of inhibition zone, statistical analysis showed that inhibitory effects of supernatants significantly decreased by increasing temperature with *p*-value = 0.0215.

The final pH of supernatant from *Enterococcus* culture stabilized at 4.5 (± 0.1) after 3 days of incubation. Following pH adjustment to different values, supernatants retained activity within a pH range of 2.5—9.5. Notably, the culture exhibited higher activity at acidic pH levels (2.5, 3.5, and 5.5), with inhibition zones reaching about 10 mm, compared to alkaline pH levels (8.5 and 9.5), where the diameter of the inhibition zone was nearly 5 mm (Fig. 3). These observations were confirmed by statistical analysis of data, as increasing pH level of supernatants significantly decreased their activity (p-value =  < 0.0001). Uninoculated MRS (pH = 4.5) also rendered growth of the indicator with inhibition zone = 3 mm.

### Anti-Alzheimer and anti-diabetes activity of ethyl acetate extract from *Enterococcus durans*

**Fig. 4:**
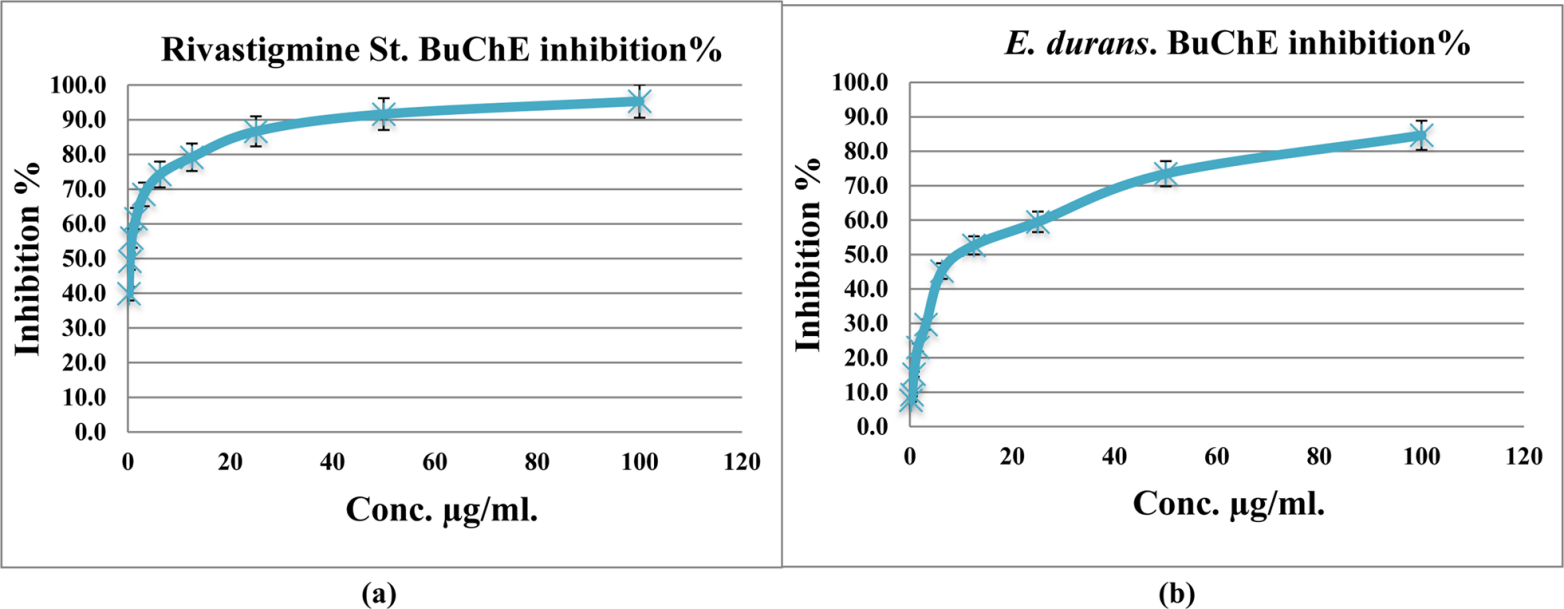
Inhibition of butyrylcholinesterase (BuCHE) by rivastigmine versus extract of *Enterococcus durans* strain AMA1. *(**a**) = Rivasigmine, (**b**) = Ethyl acetate extract of *E. durans* strain AMA1

**Fig. 5:**
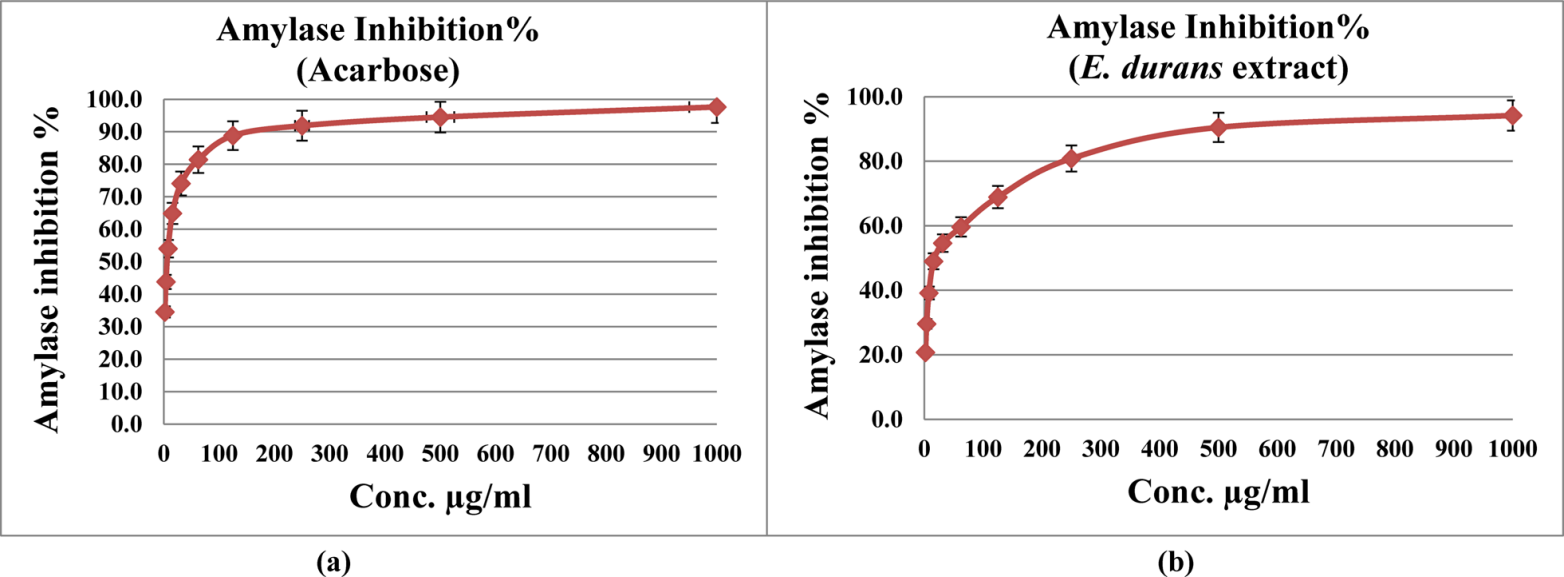
Amylase inhibition by acarbose versus extract of *Enterococcus durans* strain AMA1. *(**a**) = Acarbose, (**b**) = Ethyl acetate extract of *E. durans* strain AMA1

Butyrylcholinesterase inhibition was estimated to be 95.3% for rivastigmine and 84.6% for CFS (Fig. 4), while amylase inhibition of the medication (acarbose) and CFS was 97.6% & 94.2%, respectively (Fig. 5). IC_50_ for anti-Alzheimer activity was 9.62 µg/ml and 21.24 µg/ml for anti- diabetes activity.

### Gas chromatography–mass spectrometry (GC–MS) analysis

**Fig. 6:**
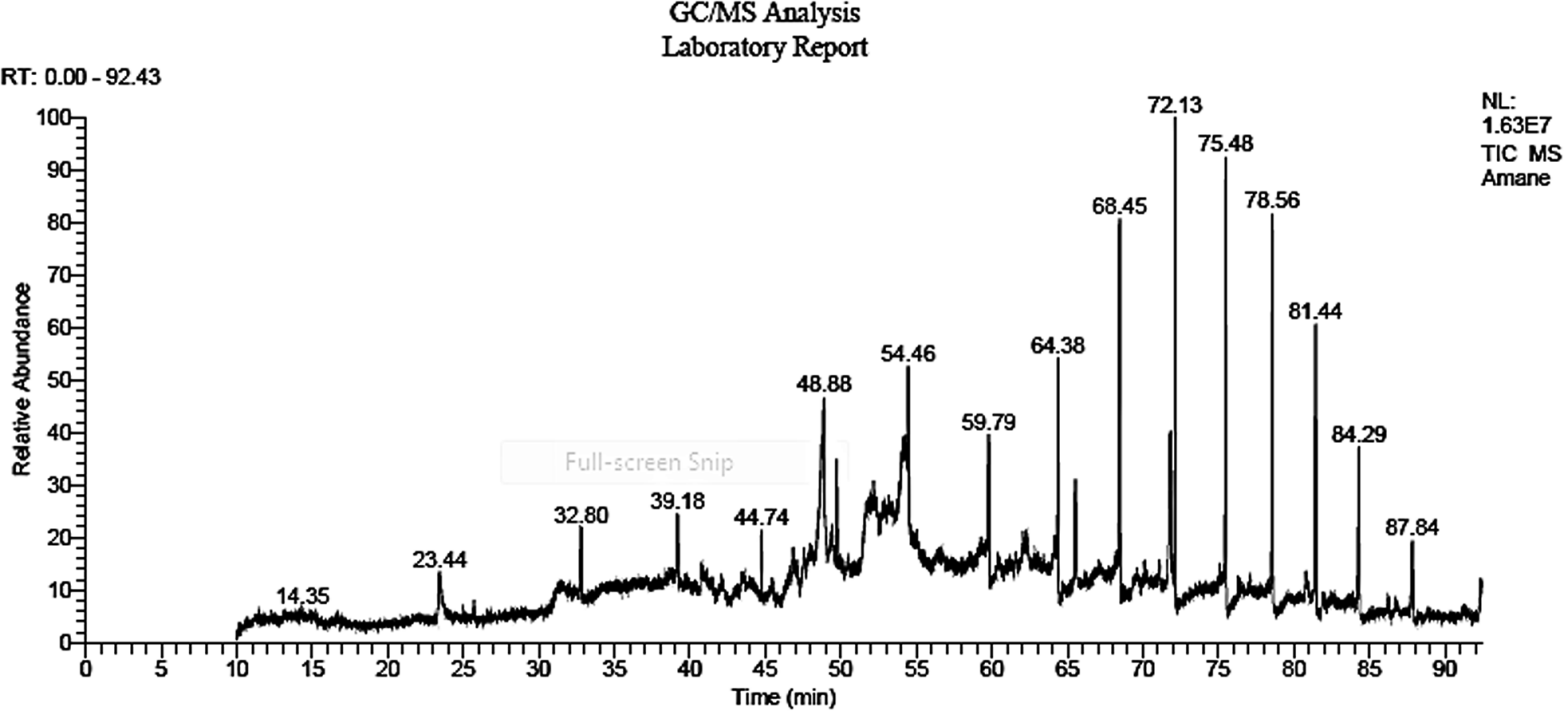
GC–MS Spectrum of ethyl acetate extract of *Enterococcus durans* strain AMA1

The most abundant metabolites in our sample were 1H-purin-6-amine, [(2-fluorophenyl) methyl]- (29.72%), hexadecanoic acid, 2,3 dihydroxypro polyester (18.60%), oleic acid (11.60%) and 9-octadecenamide (6.54%) (Table 2 and Fig. 6).

**Table 2:** Compounds identified in ethyl acetate extract from *Enterococcus durans* strain AMA1 using GC–MS technique

No	RT	Compound name	Molecular formula	Molecular weight	Area %
1	23.43	2-Methoxy-4-vinylphenol	C_9_H_10_O_2_	150	3.26
2	40.77	(2S,2′S)-2,2′-Bis[1,4,7,10,13-pentaoxa-cyclopentadecane]	C_20_H_38_O_10_	438	1.26
3	42.13	2-Aminoethanethiol hydrogen sulfate (ester)	C_2_H_7_NO_3_S_2_	157	0.847
4	43.49	Propanoic acid, 2-(3-acetoxy-4,4,14-trim ethylandrost-8-en-17-yl	C_27_H_42_O_4_	430	0.47
5	45.45	9,10-Secocholesta-5,7,10(19)-triene-3,24,25-triol, (3á,5Z,7E)-	C_27_H_44_O_3_	416	0.95
**6**	**48.87**	**Hexadecanoic acid, 2,3-dihydroxypropyl ester**	**C**_**19**_**H**_**38**_**O**_**4**_	**330**	**18.6**
7	49.39	Octaethylene glycol monododecyl ether	C_28_H_58_O_9_	538	1.95
**8**	**54.07**	**Oleic acid**	**C**_**18**_**H**_**34**_**O**_**2**_	**282**	**11.60**
9	59.26	Heptaethylene glycol monododecyl ether	C_26_H_54_O_8_	494	1.13
10	59.3	3-(2,5,8,11,14-Pentaoxacyclohexadecyl)-1,5,8,11,14,17-hexooxacyclononadecane	C_24_H_46_O_11_	510	0.252
11	60.47	3-(1,3-Dihydroxyisopropyl)-1,5,8,11,14,17-hexaoxacyclononadecane	C_16_H_32_O_8_	352	0.378
12	60.97	Hexadecadienoic acid, methyl ester	C_17_H_30_O_2_	266	0.306
13	61.54	[1,1′-Bicyclopropyl]-2- octanoic acid, 2′-hexyl-, methyl ester	C_21_H_38_O_2_	322	1.784
14	62.07	cis-Vaccenic acid	C_18_H_34_O_2_	282	1.135
15	62.29	Tetraneurin—A—diol	C_15_H_20_O_5_	280	1.33
16	62.85	Ethyl iso-allocholate	C_26_H_44_O_5_	436	0.648
17	63.07	1-Heptatriacotanol	C_37_H_76_O	536	0.937
18	63.41	7-Methyl-Z-tetradecen-1-ol acetate	C_17_H_32_O_2_	268	0.396
19	65.53	1,2-Benzenedicarboxylic acid	C_24_H_38_O_4_	390	4.41
20	69.65	Dicyclohexyl-18 crown 6	C_20_H_36_O_6_	372	1.586
21	70.10	9-Octadecenoic acid, (2-phenyl-1,3-dioxolan-4-yl) methyl ester, cis-	C_28_H_44_O_4_	444	1.063
22	70.30	9-Octadecenoic acid (z)-,2-hydroxy-1- (hydroxymethyl) ethyl ester	C_21_H_40_O_4_	356	0.594
23	71.10	4H-1-benzopyran -4-one,2-(3,4-dimethoxy phenyl)-3,5-dihydroxy-7-methoxy	C_18_H_16_O_7_	344	1.352
**24**	**71.83**	**9-Octadecenamide**	**C**_**18**_**H**_**35**_**NO**	**281**	**6.54**
**25**	**72.13**	**1H-purin-6-amine, [(2-fluoropheny l)methyl]-**	**C**_**12**_**H**_**10**_**FN**_**5**_	**243**	**29.72**
26	76.33	1-Heptatriacotanol	C_37_H_76_O	536	0.77
27	76.50	Linoleic acid ethyl ester	C_20_H_36_O_2_	308	0.955
28	77.13	12,24-Divinyl-1,13- dioxacyclotetracosane-2,14-dione	C_26_H_44_O_4_	420	0.378
29	78.27	3-Oxo-20-methyl- 11-à-hydroxyconanine-1,4-diene	C_22_H_31_NO_2_	341	0.66
30	80.81	Cholestan-3-one, cyclic 1,2-ethanediylaetal,(5á)-	C_29_H_50_O_2_	430	1.26
31	80.93	PSI.,.PSI.-caroten e, 1,1′,2,2′-tetrahydro-1,1′-dimethoxy	C_42_H_64_O_2_	600	0.793
32	81.9	18,19-Secoyohimban-19-oic acid, 16,17,20,21-tetradehydro-16-(hydroxyl methyl)-, methyl ester, (15á,16E)-	C_21_H_24_N_2_O_3_	352	0.83
33	81.99	Trideuteriomethyl 10-epoxy-7-ethyl- 3,11-dimethyltri deca-2,6-dienoate	C_18_H_27_D_3_O_3_	297	0.46
34	82.47	7,8-Epoxylanostan-11- ol, 3-acetoxy-	C_32_H_54_O_4_	502	0.45
35	86.25	Celidoniol, deoxy	C_29_H_60_	408	0.9

*Compounds indicated in bold, represent the major constituents in *E. durans* strain AMA1 extract

## Discussion

### Isolation and identification of *Enterococcus* sp.

Lactic acid bacteria (LAB) are a well-defined group of Gram-positive, non-spore-forming, and catalase-negative bacteria known for their ability to produce acids from glucose. The identification of enterococci, a subset of LAB, was crucial in our study. Our isolate displayed characteristics consistent with *Enterococcus*, such as Gram-positive cocci arranged in chains, catalase negativity, and growth under homofermentative conditions (Andrighetto et al. 2001; Abanoz and Kunduhoglu 2018). The absence of gas production ruled out membership in heterofermentative genera like *Leuconostoc* or *Weisella*.

Sequencing of 16S rRNA gene validated the identification of the target isolate as *Enterococcus*. Within the genus *Enterococcus*, our isolate was identified at the species level, as *Enterococcus durans*. The versatility of *Enterococcus* was evident in its isolation from diverse sources, including milk, clinical materials, food, and the environment (Morandi et al. 2012; Yerlikaya and Akbulut 2020). Many species, such as *E. lactis*, *E. durans*, and *E. hirae*, were discussed, highlighting the variations in their acidification abilities for different substrates. The genomic identification through 16S rRNA sequencing aligned seamlessly with the biochemical characterization, reinforcing the accurate identification of the isolate as *Enterococcus durans (E. durans)*. The deposition of isolate in GenBank further enhances the reliability of our findings.

### Antimicrobial activity of *Enterococcus durans*

Many species of probiotics have been reported to acquire beneficial effects on human health: *Lactobacillus (L. plantarum, L. rhamnosus, L. acidophilus), Lactococcus (L. lactis), Pediococccus (P. acidilactici), Streptococcus (S. thermophilus), Bifidobacterium (B. infantis, B. breve, B. longum), Enterococcus (E. durans, E. faecium)* (Anand et al. 2022). Our isolate demonstrated significant inhibition and a broad spectrum of antibacterial activity against Gram-positive bacteria like *S. aureus*, *B. subtilis* and *E. faecalis* and Gram negative ones (*Escherichia coli, Klebsiella pneumonia* and *Salmonella typhi*). This finding aligns with previous researches indicating the antimicrobial potential of LAB against various pathogens, showcasing their role as probiotics and biopreservatives (Gaaloul et al. 2015; Abanoz and Kunduhoglu 2018; Abesinghe et al. 2020). The antibacterial activity of enterococci was demonstrated by many researchers (Javed et al. 2011; Ahmadova et al. 2013; Han et al. 2014). *Enterococcus faecalis* and *Enterococcus hirae* showed antimicrobial property against both gram positive indicator (*S. aureus* ATCC 25923) and gram negative one (*E. coli* ATCC 25922) (Sonbol et al. 2020).

Sensitivity of the indicator bacteria (*Staphylococcus* sp.) to acidic uninoculated MRS medium at pH (4.5)- which is the final pH of our strain- means that organic acids in our sample are one of the possible interpretations of antibacterial activity. However, inhibition caused by supernatant of this strain was at least three times more than acidic uninoculated MRS, which means that other antimicrobial components were found in the sample. The antagonistic mechanism between LAB and harmful genera relies on the production of metabolites, including organic acids (such as lactic and acetic acid, leading to decrease in pH that is unfavorable to some pathogens and spoilage microorganisms), bacteriocins, hydrogen peroxide, antifungal peptides, and competition for nutrients (Vasiljevic and Shah 2008; Rahmeh et al. 2019). Bacteriocins from LAB have been broadly used as biopreservatives (e.g.: Nisin), to control pathogenic bacteria in food products including cheese (Khelissa et al. 2021). Several strains of *Enterococcus* are applied as starter cultures (Moreno et al. 2006), and some are used as probiotics (Holzapfel et al. 2018). Eloff (2021), categorized MIC values as the following: outstanding activity ≤ 20 µg/ml, excellent activity 21–40 µg/ml, very good activity 41–80 µg/ml, good activity 81–160 µg/ml, average activity 161–320 µg/ml, and weak activity > 320 µg/ml (Eloff 2021)**.** Based on these recommendations, the activity of *E. durans* extract in the current study ranges from outstanding (against *Bacillus subtilis*, *Staphylococcus aureus*), excellent (*Enterococcus faecalis*, *Salmonella typhi*) to very good (*Escherichia coli, Klebsiella pneumoniae*).

The GC- MS analysis of the ethyl acetate extract of our bacterial *Enterococcus durans* strain, showed the presence of different fatty acids and their derivatives like oleic acid. Previous studies mentioned that fatty acids contribute to the antibacterial activity. It was reported that OH groups of fatty acids affected the cell membrane of bacteria (Wojtczak et al. 1999).

The cell-free supernatant (CFS) from our isolate demonstrated remarkable stability across a wide pH range (2.5–9.5) and even after autoclaving. This robust stability is consistent with previous studies on LAB-derived bacteriocins, supporting their potential application as natural preservatives (Lü et al. 2014). The ability of our isolate to maintain antimicrobial activity under extreme conditions enhances their appeal for various industrial applications.

### Anti-Alzheimer and anti-diabetes activity of *Enterococcus durans*

Gradual loss of brain cells and the accumulation of amyloid β (Aβ) plaques outside neurons, occur in brains of Alzheimer’s patients (Qu et al. 2024). This is related to a deficiency in the enzyme responsible for acetylcholine (ACh) level. The cholinsterase terminates the effects of neurotransmitters hydrolyzing ACh to choline and acetate in the brain. Therefore, cholinesterase inhibitors are applied as treatments for Alzheimer’s disease (Vladimir-Knežević et al. 2014; Lockridge 2015). Inhibition of butyrylcholinesterase (BuChE) by our lactic *Enterococcus durans* isolate compared with the drug rivastigmine, wasn’t far. Recently, some published results announced the role of probiotics to control dementia and Alzheimer. One of the bioactive strains that accumulate a high yield of short-chain fatty acids is *Enterococcus* (DM9112). Mices-treated with that strain showed richness of intestinal microbiota, increased colonization of beneficial bacteria, and reduced neuroinflammation (Li et al. 2023). Administration of *Lactobacillus* and *Bifidobacterium* species significantly improved the memory deficit in AD model mice, and also inhibits the AD-related pathological mechanisms (Athari Nik Azm et al. 2018). Probiotics (*Lacticaseibacillus rhamnosus* HA-114 or *Bifidobacterium longum* R0175) applied for 12 weeks reduced oxidative stress and inflammation in patients with mild and moderate AD (Akhgarjand et al. 2024). Rats treated with probiotics, significantly had reduced number of dead cells in the brains compared with the AD group. *Bifidobacterium bifidum* and *Lactobacillus plantarum* combined with exercise training can improve spatial learning impairment in the AD rats. Exercise and probiotics lead to upregulating acetylcholine (ACH) in AD rats (Shamsipour et al. 2021).

The α–amylase is one of the major products of salivary glands and pancreas that plays a pivotal role in the digestion of starch and glycogen (Brayer et al. 2000). Inhibiting activities of carbohydrate digestion enzymes involving amylase is applied to delay glucose absorption in diabetes patients. Our lactic *Enterococcus durans* strain has the activity of inhibiting the enzyme amylase that was similar to the effect of the medication (acarbose). Several studies have revealed that LAB have α-glucosidase inhibition activities. Exopolysaccharides produced by LAB are behind this activity (Lee and Kim 2019; Ayyash et al. 2020). Extract of *Artemisia capillaris* fermented with the probiotic *Leuconostoc mesenteroides,* had a significant impact on α-glucosidase inhibition (anti-diabetes), and maintained acetyl- and butyryl cholinesterase inhibitory activity (anti-Alzheimer) (Yoon and Kim 2022).

### Gas chromatography and mass spectrometry (GC–MS) analysis

This technique is a helpful tool to identify general chemical profile of bioactive extracts (Iordache et al. 2009). The major components of the extract of our lactic acid bacterium *Enterococcus durans* strain AMA1 weren’t previously reported form *Enterococcus durans,* but bioactivity of these constituents (antimicrobial, anti-Alzheimer and anti-diabetes) from plant sources and other bacterial species was recently documented. The most common here was 1H-Purin-6-amine, [(2-fluorophenyl) methyl]-(CAS) (29.7% of our sample). It is reported for its antimicrobial activities and is subsequently highlighted as a potent inhibitor of several enzymes like acyl coenzyme A, cholesterol acyltransferase and heat shock protein 90. Its derivatives are also known to possess anti-inflammatory, antitumor, antiulcer activity. Molecular docking studies of GC–MS compounds for the inhibition of α-amylase activity revealed that (1H-Purin-6-amine, [(2-fluorophenyl) methyl]-(CAS), achieved good enzyme inhibition scores (Akshatha et al. 2021).

The second abundant metabolite in the target *Enterococcus durans* sample is the fatty acid ester namely hexadecanoic acid, 2, 3-dihydroxypropyl ester that resembles (18.6%). This compound is known with its antibacterial and anticancer properties (Moni et al. 2021). Oleic acid that constitutes (11.6%) of our sample, is a mono-saturated fatty acid abundant in olive fruits. It is vital to cell membrane, circulatory system and healthy brains. Depressive disorders and Alzheimer diseases are correlated to decreased levels of oleic acid. A significant decrease in oleic acid has been noticed in the brains of Alzheimer’s patients and those with major depressive disorders (Santa-María et al. 2023). 9-octadecenamide (*Z*) (6.54%) is an oleic acid amide that has a strong antioxidant, antibacterial, anti-inflammatory, and hypolipidaemic properties (Cheng et al. 2010). Dos Reis et al. (2019) found 75.83% of 9-octadecenamide in the ethyl acetate extract of the fungus *Diaporthe schini*, was active against *Staphylococcus epidermidis,* *Enterobacter aerogenes* and *Klebsiella pneumoniae*(Dos Reis et al. 2019). The antibacterial action detected in organic extracts from plants like *Leucosidea sericea* and *Searsia lancea* was attributed to 9-octadecenamide (Z) (Makhubu et al. 2023).

Challenges regarding application of probiotis are discussed recently. It have been shown that some medications interfere with the viability of the probiotics and inhibit their therapeutic potential (Todorov et al. 2011; Jeronymo-Ceneviva et al. 2014). As a result, a loss of probiotic effectiveness and proliferation of pathogenic bacteria may occur, gut clinical disorders, and inflammatory diseases (Vinderola et al. 2005; Galdeano et al. 2007).

*In conclusion*, our strain *Enterococcus durans* strain AMA1 has promising features, including tolerance to bile salts and acidity, potent antibacterial activity and exceptional thermal stability of cell-free supernatant (CFS). Exopolysaccharides noticed on solid medium with high concentration of sugar, are applied in fermented milks to improve their texture and manufacture of low-fat cheeses (mozzarella). The application of both traditional biochemical tests and molecular tools, such as 16S rRNA sequencing, ensured accurate identification. The strain was deposited in GenBank and available for further investigations at the Culture Collection Ain Shams University (CCASU) of the World Data Centre for Microorganisms (WDCM) under specific code (10.12210/ccinfo.1186). This study establishes that *E. durans* strain AMA1 from yoghurt is a rich reservoir of biologically active compounds with antimicrobial, anti-Alzheimer and anti-diabetes efficiency. The strain holds potential as a natural preservative, contributing to the quest for safer and more sustainable alternatives to chemical preservatives in the food industry. To the best of our understanding, this is the first work identifying 1H-Purin-6-amine, [(2-fluorophenyl) methyl]-(CAS), 2,3-dihydroxypropyl hexadecanoate, oleic acid and 9-octadecenamide from *E. durans.*

## Data Availability

The datasets utilized and/or examined in the present study can be obtained by contacting the corresponding author. Additionally, the genetic sequence of the strain analyzed has been submitted to the GenBank nucleotide sequence database at the National Library of Medicine, National Center for Biotechnology Information (NCBI). The assigned accession number for the sequence is OP648139 (https://www.ncbi.nlm.nih.gov/nuccore/OP648139.1?report=GenBank).

## Abbreviations

LAB: Lactic acid bacteria
MRS: de Man, Rogosa and Sharpe culture medium
CFSs: Cell-free supernatants
SDW: Sterile distilled water
GRAS: Generally Recognized as Safe
EPS: Exopolysaccharides
AD: Alzheimer’s disease
BuChE: Butyrylcholinesterase
